# Identification of an Metabolic Related Risk Signature Predicts Prognosis in Cervical Cancer and Correlates With Immune Infiltration

**DOI:** 10.3389/fcell.2021.677831

**Published:** 2021-06-24

**Authors:** Chunliang Shang, Jiaming Huang, Hongyan Guo

**Affiliations:** ^1^Department of Obstetrics and Gynecology, Peking University Third Hospital, Beijing, China; ^2^Department of Obstetrics and Gynecology, The First Affiliated Hospital, Sun Yat-sen University, Guangzhou, China; ^3^National Clinical Research Center for Obstetrics and Gynecology, Beijing, China

**Keywords:** prognosis, metabolic signature, P4HA2, immune infiltration, lipid droplets, cervical cancer

## Abstract

The tumor metabolic reprogramming contributes to the progression and prognosis of cervical cancer (CC). However, the potential remodeling mechanisms of tumor metabolism in the immune microenvironment of CC remain largely unknown. In this study, we first performed microarray analysis to identify differential metabolic gene expression. A novel 5-metabolic-related genes (MRGs) signature comprising P4HA1, P4HA2, ABL2, GLTP, and CYP4F12 was established to better predict prognosis of CC using LASSO-Cox regression analysis. This signature could reveal the metabolic features and monitor the immune status of tumor microenvironment (TME). Among them, P4HA2 was significantly upregulated in CC tissues and negatively correlated with CD8+T cells. Knockdown of P4HA2 inhibited lipid droplets (LDs) accumulation and cancer cells invasion. Moreover, P4HA2 knockdown significantly suppressed PD-L1 expression. This study provides a new and feasible method for evaluating the prognosis of CC and explores the potential value to navigate metabolic pathways to enhance anti-tumor immunity and immunotherapy.

## Introduction

Cervical cancer (CC) is the]aqvskip-2pc top two cause of cancer death affecting women worldwide (Siegel et al., 2020). Due to limited therapeutic options, the 5-year overall survival (OS) of CC patients with recurrent, persistent and metastatic is only about 15% (Liu et al., 2019). As an increasing understanding of tumor microenvironment (TME), immunotherapy has been a promising approach to advanced/metastatic CC (Attademo et al., 2020). How to screen the patients who can benefit from immunotherapy and increase the sensitivity of the chemotherapy, is a hot topic and needs to be further explored.

The reprogramming of energy metabolism which an emerging hallmarks of cancer, is well-established prominent hallmarks of cancer (Hanahan and Weinberg, 2011). Interestingly, growing evidence indicates that the fatty acid (FA) metabolism confers a selective advantage for tumor metastatic process (Nath and Chan, 2016; Pascual et al., 2016). Previous reports have identified that the metabolism of tumor cells could affect the metastasis to improve patient survival (Shang et al., 2018). Moreover, tumor cells and immune cells experience metabolically completion, modulating antitumor immune responses (Li et al., 2019). Lipid droplets (LDs) are regarded as cytoplasmic organelles within tumor cells and immune cells to store lipids, which play an important role in modulating cancer metasatsis (Shang et al., 2020). Thus, Targeting metabolic pathways in terms of anticancer immunotherapy are important directions for the treatment of tumors.

In this study, a novel independent prognostic model based on metabolic-related gene (MRG) signature was identified and constructed by microarray analysis and The Cancer Genome Atlas (TCGA) database to reflect the tumor immune status in CC. Additionally, nomograms associated with MRGs were established. This prognostic model could delineate the metabolic features of CC and further monitor the status of immune infiltration. Surprisingly, P4HA2 which was included in this prognostic risk model, could promote the LDs accumulation in CC cells and exhibited a close relationship with the abundance of different cell types in the TME and immune checkpoint blockade. Our findings manifested that these prognositic MRGs system play crucial roles in patient management and provide potential drug targets to develop effective immunotherapies for CC.

## Materials and Methods

### Patients and Sample Collection

This study was conducted in accordance with the Declaration of Helsinki. Each participant signed an informed consent form, and the Ethical Committee of the Peking University Third Hospital approved this study. Five CC tissues and six normal cervical tissues were collected for mRNA microarray. Another 15 normal or 15 CC tissues were selected for validation. All freshly frozen CC tissues were collected from the Peking University Third Hospital (Beijing, China). All of the cases were histologically confirmed by pathologists and no patients had received chemotherapy or radiotherapy prior to surgery. The normal controls were collected from women who underwent hysterectomy for non-malignant conditions. All tissue specimens were immediately frozen at −80°C.

### Cell Culture

The human CC cell line SiHa and HeLa was purchased from the American Type Culture Collection (Manassas, VA, United States) and cultured in Dulbecco’s Modified Eagle’s medium (Gibco; Thermo Fisher Scientific, Inc.) in a humid atmosphere with 5% CO2 at 37°C. Authenticity of SiHa and HeLa cell lines was verified by using short tandem repeat (STR) genotyping.

### Data Analysis of Metabolic-Related mRNA Expression Profiles

The differentially mRNA expression profiles were analyzed by Agilent Feature Extraction software (version 11.0.1.1). A comprehensive MRGs set was extracted from the Metabolic Atlas in the Human Protein Atlas (HPA) database. Differentially expressed metabolic-related mRNAs were screened with *p* < 0.05 and —fold change| > 1.5.

### RNA Extraction and Quantitative Real-Time PCR

Total RNA was extracted using TRIzol reagent (TaKaRa, Japan). Quantitative real-time PCR (qRT-PCR) was performed with SYBR Premix Ex Taq (TaKaRa, Dalian, China) using the 7500 Fast Real-Time PCR system (Applied Biosystems, United States). GAPDH was utilized as an internal standard control. The gene-specific primers were as follows: P4HA2, forward 5′-AGTACCAGGCAATGCTGAGT-3′, reverse 5′-CCTCTTCTGTCTACGGGGTG-3′; PD-L1 forward, 5′-TGGCATTTGCTGAACGCATTT-3′, reverse, 5′-TGCAGCCAGGTCTAATTGTTTT-3′; GAPDH, forward 5′-CCTGTTCGACAGTCAGCCGCAT-3′, reverse 5′-GACTCC GACCTTCACCTTCCCC-3′. Relative RNA expression levels were calculated by the relative quantification method (2^–ΔΔCT^).

### Cell Transfection

SiRNAs targeting P4HA2 and a negative control were provided by GeneCreate (Wuhan, China). The following oligonucleotide against genes were used: siRNA against P4HA2 (5′-CGAGATACTTTCAAGCATTTA-3′). Transfection was conducted using Lipofectamine RNAiMAX Reagent (Invitrogen, United States) according the manufacturer’s recommended protocol.

### Quantification of Lipid Droplets Accumulation

For cell LD quantification, the lipophilic fluorescence dye BODIPY 493/503 (Invitrogen) was employed to monitor LD accumulation in SiHa cells. Cells grown on coverslips were fixed with 4% paraformaldehyde and stained with BODIPY 493/503 (1 μg/ml) for 45 min. Nuclear recognition was based on DAPI staining (1 μg/mL). Fluorescence was analyzed by Olympus FV-1000 confocal microscope (Olympus, Tokyo, Japan).

### Transwell Assays

2 × 10^5^ cells in 100 μl serum-free medium were loaded in the upper chamber pre-coated with 50 μl Matrigel (BD Biosciences). Medium containing 10% bovine serum albumin were added to the lower chambers. After incubation, cells which adhered to the lower surface were fixed and stained. Invasiveness was determined by counting cells in five randomly selected visual fields.

### Data Collection

After excluding patients with missing expression information, the RNA expression data containing corresponding clinical information of CC, were downloaded from the TCGA database, including 303 CC tissues (Supplementary Table 1)^1^.

### Identification and Construction of the Metabolic Gene Signature

Genes with *P* values less than 0.0001 in maximally separated Kaplan–Meier analysis were defined as prognostic genes. Then, a least absolute shrinkage and selection operator (LASSO) regression was performed to picks the optimal number of potential metabolic genes to build a prognostic MRGs model. Finally, the Risk Score of each sample was calculated based on the LASSO-Cox regression co-efficiency through the following formula: $\text{Risk score }=\;\sum_{i=1}^{n}Coef_{i}\;{\;}^{*}X_{i}$, *Coef*_i_ is the risk coefficient of prognostic MRGs and X_i_ is the expression value. Then the optimal cutoff value was determined by the “sur_cutpoint” function of the “survminer” R package. It automatically calculated the segmentation point with the minimum *P* value. Patients were divided into Low- and High-risk groups according to the best cutoff values or median values.

### Development and Evaluation of the Nomogram

Plotted nomogram calibration curves based on the proper MRGs determined by univariate and multivariate cox regression analysis through “rms” R package. The forest was used to show the *P* value, HR and 95% confidence interval (CI) of each variable through “forestplot” R package.

### Correlation Analysis Between MRGs Model and Tumor Microenvironment Scores

The ESTIMATE algorithm was employed to calculate the microenvironment scores of tumors (including stromal, immune, and TME scores) based on expression data from TCGA database^2^ in the CC tissues. ESTIMATE algorithm is based on single-sample gene set enrichment analysis. For each sample from TCGA database, the gene expression values were normalized and sorted by rank. The empirical cumulative distribution functions of each gene and the other genes in the signature were calculated based on absolute expression rather than differential expression. Then, the microenvironment score is automatically output (Yoshihara et al., 2013). We compared the differences between the three kinds of scores in the high- and low-MRG risk model groups, and explore their impact on the prognosis of CC considering the best cutoff values possessed by three kinds of scores.

### Analysis of the Relationship Between Immune Infiltration and MRGs

CIBERSORT is a versatile computational method for quantifying cell fractions from bulk tissue gene expression profiles (Newman et al., 2015). We estimated the fraction of the 22 subtype immune cells in CC patients from TCGA database through the CIBERSORT algorithm to extend the utility of this MRGs signature^3^. The differences in immune cell infiltration were also evaluated by Tumor Immune Estimation Resource (TIMER^4^) including CD4+T cells, B cells, CD8+T cells, neutrophils, macrophages, and dendritic cells. The correlations between hub MRGs expression and the abundances of immune infiltrates, immune checkpoint molecules were confirmed by TIMER database.

### Bioinformatic Analysis

A part of bioinformatic analysis including heatmap, volcano plot, gene set enrichment analysis (GSEA) and correlation coefficient matrix was performed using the OmicStudio tools at https://www.omicstudio.cn/tool. The pan-cancer analysis of immune infiltrates by xCell (Aran et al., 2017) and the different expression of hub MRGs in CC tissues and normal tissues was drawn at www.aclbi.com. All data of normal tissue samples were obtained from GTEx V8 release version^5^. Genetic alterations analysis, mainly mutations and copy number alterations, were performed on the cBioportal platform^6^. The protein expression of MRGs, as reflected by antibody staining, was investigated using the Pathology Atlas portal in the HPA database (Uhlen et al., 2015, 2017; Thul et al., 2017).

### Statistical Analysis

All statistical analyses were performed using the SPSS software package (version 13.0) and Prism 5.0 software (GraphPad Prism, Inc., La Jolla, CA, United States). The Kaplan–Meier (KM) method was used for disease-free survival (DFS) and OS analysis. Two-tailed Student’s *t*-test was used for the comparison of two independent groups. Multiple statistical packages were used in R software (version 4.0.2, R Core Team, Foundation for Statistical Computing, Vienna, Austria) to download TCGA data and generate forest plots, survival curves and the receiver operating characteristic (ROC) curve. *P*-value < 0.05 was considered significant.

## Results

### Identification of Differentially Expressed MRGs

**FIGURE 1:**
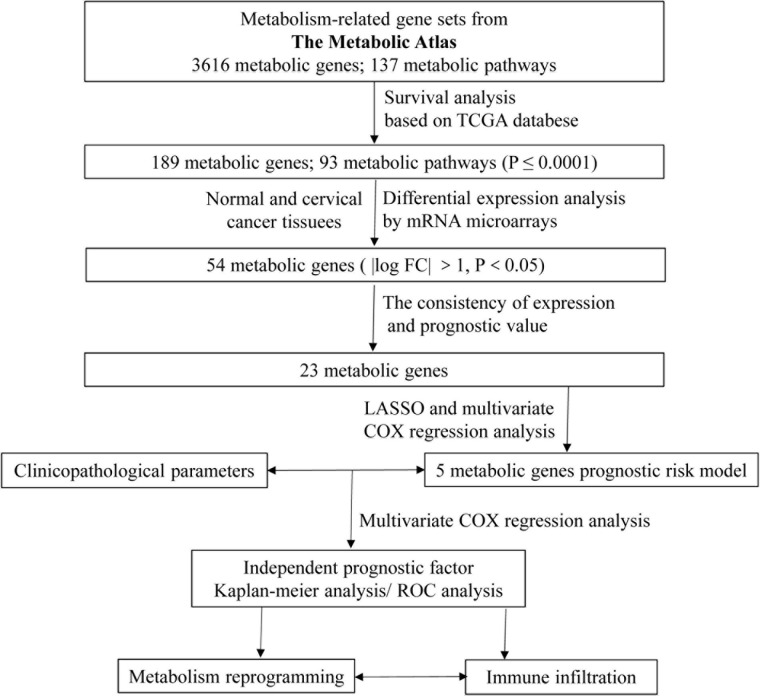
Schematic overview of the whole study.

**FIGURE 2:**
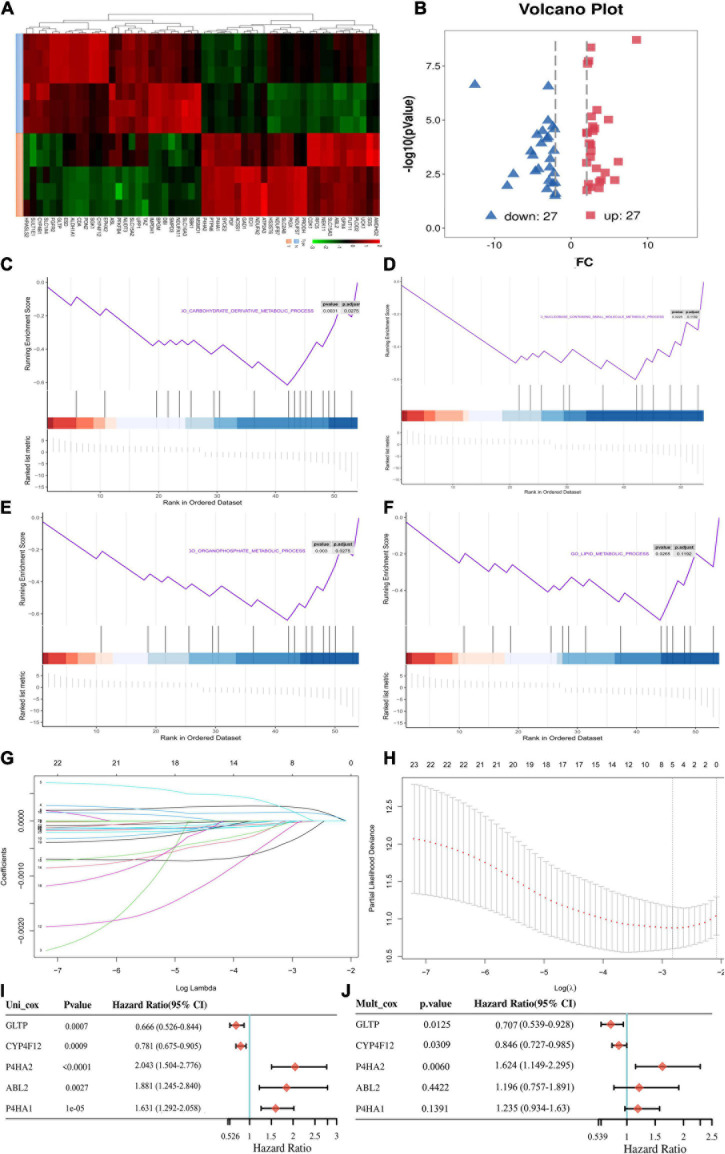
Acquisition of differentially expressed MRGs and construction of the metabolic prognostic signature. **(A)** Clustered heatmaps to explain the different expressions between CC and normal tissues. **(B)** The volcano plot of the 54 differentially expressed metabolic mRNAs. Red dots represent upregulated mRNAs, and blue dots represent downregulated mRNAs. **(C–F)** Gene set enrichment analysis (GSEA) results of significant metabolic-associated biological processes. **(G)** The LASSO was utilized to validate the parameter selection adjustment. **(H)** The distribution of LASSO coefficient profiles for metabolic model as prognostic factors. Forest plots of hazard ratios of survival-associated MRGs obtained using univariate **(I)** and multivariate **(J)** Cox regression analysis.

A flowchart was graphed to describe our study more visually in Figure 1. To identify genes involved in metabolism that affect the prognosis of CC, the MRGs sets including 3,616 MRGs and 137 metabolic pathways were firstly analyzed using KM survival analysis. A total of 189 MRGs include in 93 metabolic pathways were defined as prognostic MRGs. Then, we identified the profiles of differentially expressed MRGs between CC and normal cervical tissues (Figure 2A). There were 23 differentially expressed MRGs, containing 17 downregulated and six upregulated genes, were confirmed by consistency analysis between gene expression and prognostic value (Figure 2B). The results of GSEA analysis confirmed that the differential MRGs were related to carbohydrate derivative metabolic process, nucleobase containing small molecule metabolic process, organophosphate metabolic process and lipid metabolic process (Figures 2C–F).

### Identification of the Prognostic MRGs Model

**FIGURE 3:**
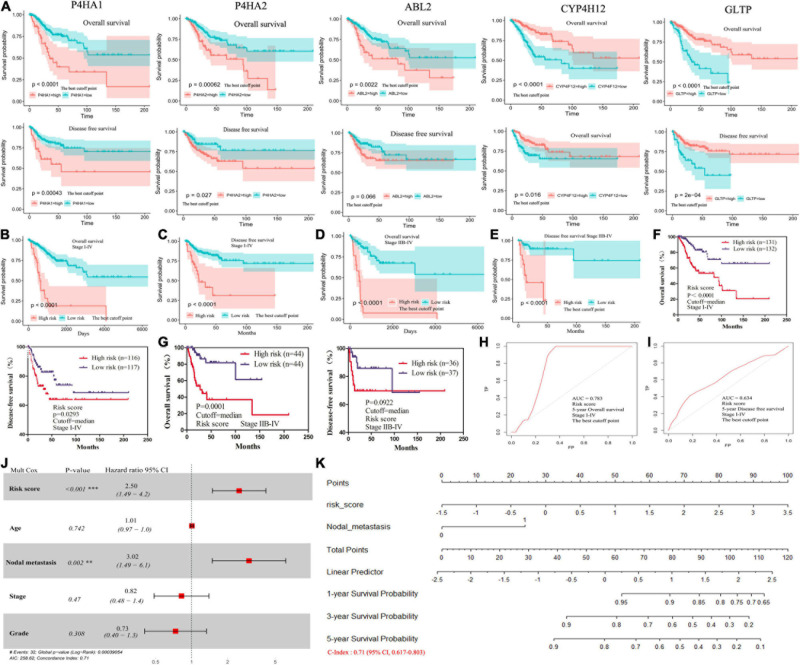
The survival analysis for 5-MRGs and the nomograms for the prediction of OS. **(A)** KM curves for OS (top), and DFS (bottom) in patients from TCGA database. **(B,C)** KM curves of OS and DFS for patients with stage I-IV divided by the best cutoff values. **(D,E)** KM curves of OS and DFS for patients with stage IIB-IV divided by the best cutoff values. **(F,G)** KM curves of OS and DFS for stage I-IV or stage IIB-IV patients divided by the median values. **(H,I)** ROC curve shows the sensitivity and specificity of MRGs model for predicting OS and DFS. **(J)** The forest plots of the correlation of clinical features and MRGs model with OS. **(K)** Nomogram for predicting the 1-, 3- or 5-year OS.

The differentially expressed prognostic MRGs were analyzed in a LASSO-Cox regression model (Figures 2G,H). Finally, five candidate prognostic MRGs signature were identified, namely, Prolyl 4-Hydroxylase Subunit Alpha 2 (P4HA2), Prolyl 4-Hydroxylase Subunit Alpha 1 (P4HA1), ABL Proto-Oncogene 2 (ABL2), Cytochrome P450 Family 4 Subfamily F Member 12 (CYP4F12), and glycolipid transfer protein (GLTP) (Figures 2I,J). Through KM survival analysis, we further identified that high expression of P4HA2, P4HA1 and ABL2 with best cutoff value or median value were unfavorable in CC; however, CYP4F12 and GLTP were favorable prognostic factors for survival (Figure 3A and Supplementary Figure 1A).

Then, we calculated a prognostic risk score with the following formula: Risk Score = [P4HA2 × (0.485)] + [P4HA1 × (0.211)] + [ABL2 × (0.179)] + [CYP4F12 × (−0.167)] + [GLTP × (−0.347)] (Table 1). The patients were divided into high-risk and low-risk groups according to the best cutoff values. The KM analysis showed that patients (stage I–IV) in the high-risk group had worse OS and DFS than patients in the low-risk group (Figures 3B,C and Supplementary Figure 1B). All patients with advanced-stage (IIB-IV) were further evaluated by the risk score. Similar significant predictive power of the 5-MRGs signature was found in both OS (*n* = 109) and DFS (*n* = 87) (Figures 3D,E and Supplementary Figure 1C). At the same time, OS was also significantly worse in the high-risk group with the median cutoff value (Figures 3F,G). Significantly, the overall 5-year survival rate was 47.548% /19.017 in high risk group vs. 81.400%/78.739% in low risk group in all stage patients; for advanced-stage (IIB-IV) patients, the overall 5-year survival rate was 35.997%/7.856% in high risk group vs. 76.977%/67.389% in low risk group (Table 1). Additionally, the area under the ROC curve (AUC) value of 0.783/0.737 indicated great specificity and sensitivity of the MRGs signature in predicting OS for patients with CC (Figures 3H,I and Supplementary Figure 1D). As shown in Table 1, the negative predictive values of MRGs model for DFS was 88% and the negative predictive values for OS was 59%. Taken together, these data may provide a powerful candidate prognostic MRGs signature model to serve as a promising biomarker for predicting outcomes in CC patients.

**TABLE 1:** Clinical significance of the 5-metabolic gene signature-based risk score in predicting prognosis of patients in cervical cancer.

**Gene**	**LASSO**	**Coefficient**	**Risk score**	**Stage**	**Index**	**Cutoff**	**High risk cases**	**Low risk cases**	**5-Year survival rate**		**P-value**	**PPV%**	**NPV%**
									**High risk**	**Low risk**			
P4HA2	0.0002338408	0.485		I-IV	OS	Median	152	152	47.548	81.400	<0.0001	34	86
P4HA1	0.0001516853	0.211		I-IV	DFS	Median	131	132	60.571	75.619	0.0367	23	84
ABL2	0.00009150268	0.179		I-IV	OS	Best	47	257	19.017	78.739	<0.0001	46	81
CYP4F12	−0.0002584956	−0.167		I-IV	DFS	Best	47	216	35.117	75.405	<0.0001	23	88
GLTP	−0.00001773142	−0.347		IIB-IV	OS	Best	19	90	7.856	67.389	<0.0001	59	84
Risk score = Gene expression × Coefficient				IIB-IV	DFS	Best	20	67	24.838	89.165	<0.0001	44	88
				IIB-IV	OS	Median	54	55	35.997	76.977	<0.0001	45	86
				IIB-IV	DFS	Median	43	44	61.485	87.767	0.0561	25	86

*OS, overall survival; DFS, disease free survival; Coef: coefficient; PPV, positive predictive value; NPV, negative predictive value. The best cutoff of survival analysis was calculated by R package.*

### Independent Prognostic Validation of MRGs Model and Construction of Prognostic Nomograms

To explore whether MRGs model as an independent OS predictor, offers a measurable method to estimate the prognosis of CC patients, we constructed a nomogram that contained the MRGs model and other clinical characteristics, including age, nodal metastasis, grade, and stage. The multivariate Cox regression showed high MRGs risk score (HR 2.50; 95% CI, 1.49–4.2) and nodal metastasis (HR 3.02; 95% CI, 1.49–6.1) were independent prognostic factors (Figure 3J). A nomogram including MRGs model and nodal metastasis could provide a clinically applicable method for predicting the prognosis of CC patients. The C-index was 0.71 (95% CI: 0.617–0.803) for the TCGA cohorts (Figure 3K). Thus, our model had an approximately moderate accuracy in the prognostic prediction of CC to some extent.

### Association Between the MRGs Model and TME

**FIGURE 4:**
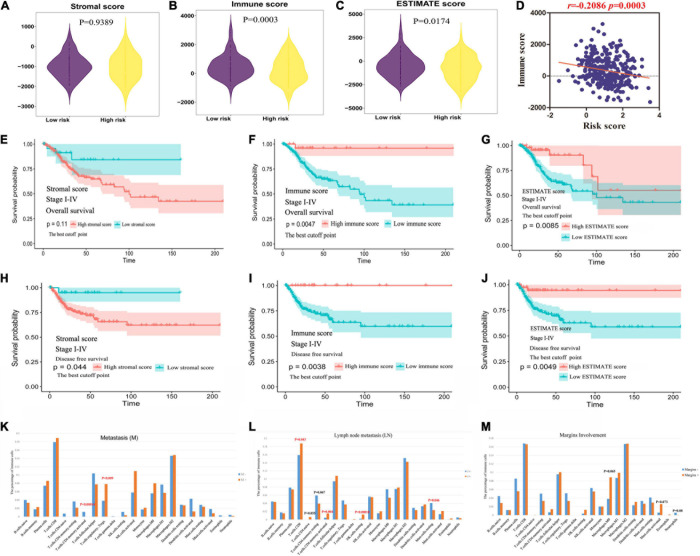
The clinical value of ESTIMATE score. **(A–C)** The relationship between immune, stromal, TME score and MRGs model. **(D)** The MRGs model were significantly negatively correlated with the immune scores. **(E–G)** The predictive value of stromal, immune and TME score in evaluating OS. **(H–J)** The predictive value of stromal, immune and TME score in evaluating DFS. **(K–M)** The immune landscape in clinicopathologic features: metastasis, lymph node metastasis, and margins involvement.

To further understand the relationship between metabolic status and TME in CC, immune score and stromal score were enrolled into the analysis. Based on the ESTIMATE method, we obtained stromal scores (range: −2586.99∼778.01) and immune scores (range: −712.91∼141.82) for all these CC patients. Both immune score and TME score of CC cases were significantly lower in high-risk group than those of low-risk cases (*P* = 0.0003, 0.0174, respectively), though no significant differences between high- and low-risk group were found for stromal scores (Figures 4A–C). The MRGs model were significantly negatively correlated with the immune scores (Figure 4D).

The predictive value of stromal/immune score in evaluating OS and DFS was explored based on TCGA database. As shown in Figures 4E–G and Supplementary Figure 1E, the OS of patients with high immune score or TME score was longer than those in low score group; however, the patients with low stromal score only showed a trend toward better OS. Consistently, patients with low immune or TME score and high stromal score was found to have significantly worse DFS (Figures 4H–J and Supplementary Figure 1F).

### The Immune Landscape in Clinicopathologic Features, Including MRGs Model

The tumor infiltrating immune cells were variously distributed in different clinicopathologic characteristics. We found that the infiltrating levels of memory activated CD4+T cells were significantly lower and regulatory Tregs cells were higher in primary tumor microenvironment (PTME) with metastasis (Figure 4K). It’s worth noting that the infiltrating levels of resting mast cells were significantly higher and CD8+T cells, memory activated CD4+T cells, resting NK cells were lower in PTME with lymph node metastasis (Figure 4L). The infiltrating levels of neutrophils cells displayed downward trend and macrophage (M0) cells, activated mast cells exhibited rising trend in the PTME with margins involvement (Figure 4M, *p* > 0.05).

**FIGURE 5:**
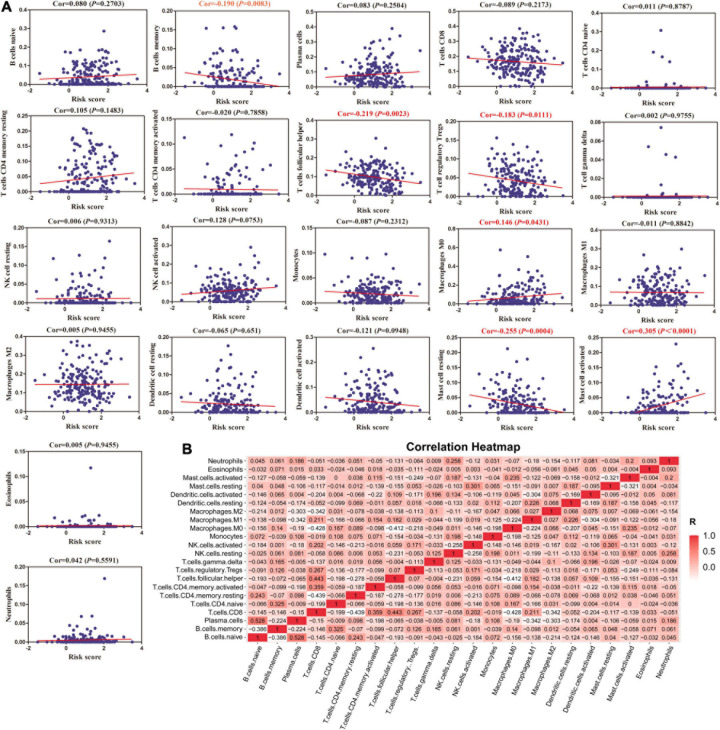
The correlation between MRGs model and immune cells. **(A)** The relationships between MRGs model and infiltration levels of 22 subtypes of immune cells calculated by CIBERSORT system. **(B)** Correlation heatmap analysis of immune cells.

Furthermore, we calculated the relationship between MRGs model and immune infiltration through CIBERSORT system. A total of six subtypes of immune cells [memory B cells, follicular helper T cell, regulatory T cells, macrophage (M0) cells, resting mast cells and activated mast cells] had an obvious negative correlation with MRGs model (Figure 5A). Meanwhile, there was a significant positive correlation between naïve B cells and plasma cells (Figure 5B).

### Validation of MRGs Expression

**FIGURE 6:**
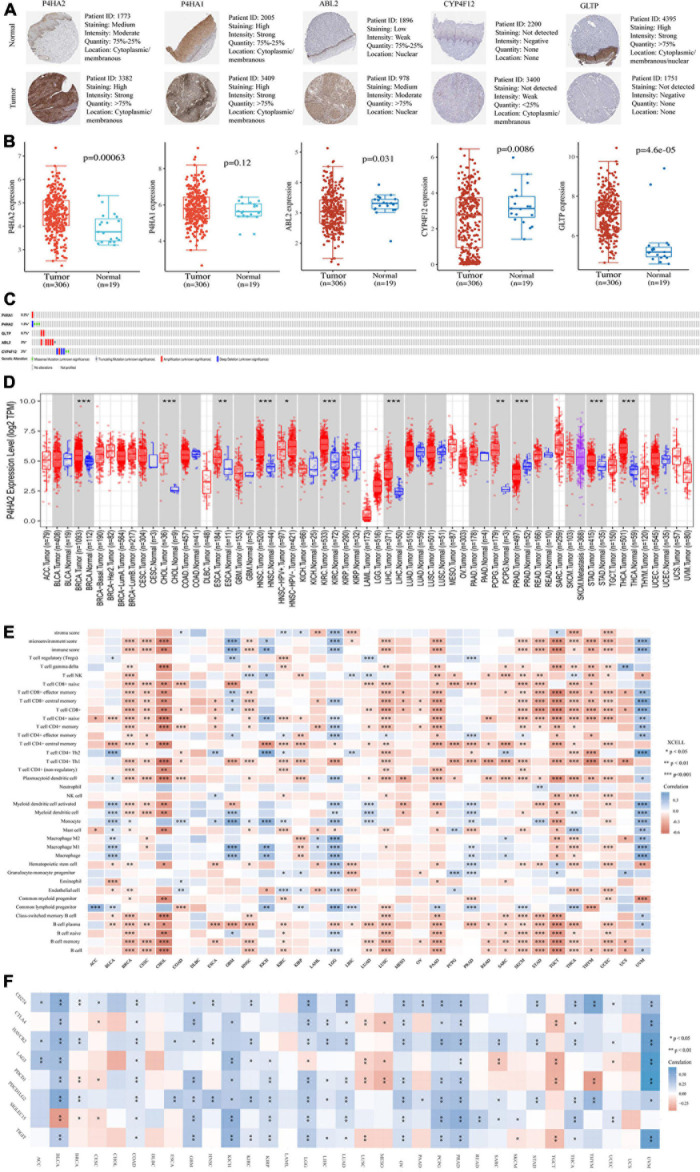
Validation of expression and pan-analysis of immune infiltration. **(A)** The representative protein expression of the 5-MRGs in CC tissues and normal tissues. **(B)** The mRNA expression levels of 5-MRGs. **(C)** Genetic alterations of the 5-MRGs in CC. **(D)** The differential expression between tumor and normal tissues for P4HA2 across all TCGA tumors. **(E)** Correlation heatmap between P4HA2 and immune infiltration across all TCGA tumors. **(F)** Correlation heatmap between P4HA2 and immune checkpoint genes across all TCGA tumors. *P < 0.05, **P < 0.01, ***P < 0.001. CNS, central nervous system; ACC, adrenocortical carcinoma; BLCA, bladder urothelial carcinoma; CESC, cervical and endocervical cancer; DLBC, diffuse large B-cell lymphoma; GBM, glioblastoma multiforme; HNSC-HPVpos, head and neck cancer-HPV positive; HNSC-HPVneg, head and neck cancer-HPV negative; KICH, kidney chromophobe; KIRP, kidney renal papillary cell carcinoma; LAML, acute myeloid leukemia; LUAD, lung adenocarcinoma; LUSC, lung squamous cell carcinoma; MESO, mesothelioma; OV, ovarian serous cystadenocarcinoma; PAAD, pancreatic adenocarcinoma; READ, rectum adenocarcinoma; SARC, sarcoma; SKCM, skin cutaneous melanoma; TGCT, testicular germ cell tumors; THYM, thymoma; UCEC, uterine corpus endometrial carcinoma; UCS, uterine carcinosarcoma; UVM, uveal melanoma.

All of the five hub prognostic MRGs were validated in protein and mRNA levels using TCGA and The HPA database. We found that P4HA2, P4HA1, and ABL2 had the higher expression levels in CC tissues than that in normal cervical tissues, while CYP4F12 and GLTP had the lower expression levels by immunohistochemistry (IHC) analysis (Figure 6A). Furthermore, the RNA-seq results showed that P4HA2, P4HA1, and CYP4F12 had the consistent expression trend, while the mRNA expression of ABL2 and GLTP in clinical sample tissues is just the opposite (Figure 6B). However, we did not observe significant expression differences of MRGs in patients with pathological staging, grade, and nodal metastasis status (Supplementary Figure 1G).

Genetic alteration are considered to be a key factor in immune tolerance breakdown (Small et al., 2018). Thus, the genetic alteration in the MRGs model was analyzed with cBioPortal software. Among them, ABL2, CYP4F12, and P4HA2 were altered in six (2%) and four (1.3%) from the 308 patients, respectively (Figure 6C). The amplification and deep deletion were the most common genetic alteration forms (Figure 6C).

### Pan-Cancer Analysis of P4HA2 Expression

P4HA2 showed consistent expression patterns at mRNA and protein levels and was an independent risk factor for evaluating patient prognosis based on the foregoing results (Figures 2J, 6B). Thus, we selected P4HA2 to compare its RNA sequencing data with that in corresponding normal tissues across cancers in TCGA using TIMER (Figure 6D). Consistent with the expression trend in CC, P4HA2 expression was upregulated in BRCA (breast invasive carcinoma), CHOL (cholangiocarcinoma), ESCA (esophageal carcinoma), HNSC (head and neck cancer), KIRC (kidney renal clear cell carcinoma), LIHC (liver hepatocellular carcinoma), PCPG (pheochromocytoma and paraganglioma), STAD (stomach adenocarcinoma), and THCA (thyroid carcinoma). The opposite trend was only observed in PRAD (prostate adenocarcinoma). Furthermore, the higher expression of P4HA2 was found in HNSC patients with HPV negative than those with HPV positive. Collectively, P4HA2 acts as a tumor promoter in most cancer types.

### Association Between P4HA2 Expression and Immune Infiltrates or Immune Checkpoint Across Cancers

It has long been recognized that immune cells are intimately associated with tumor cells. We first investigated the correlation between infiltration of immune cell subtypes and P4HA2 expression is based on the xCell across 33 cancers (Figure 6E). The results indicated that P4HA2 was notably correlated with CD8+T cells in 11 cancer types, B cells and memory B cells in 12 cancer types, plasmacytoid dendritic cells and naïve CD4+ T cells in 13 cancer types, and CD4+Th1 cells in 17 cancer types (*P* < 0.0001). Three top cancer types, BRCA, LGG (lower grade glioma) and THCA, exhibited most strongly correlation between P4HA2 with immune cells (*P* < 0.0001). Moreover, P4HA2 expression was significantly correlated with the immune score in 12 cancer types, mircroenvironment score in 14 cancer types, and stromal score in four cancer types (*P* < 0.0001). Consistent with the result in Figures 4B,D, the P4HA2 expression are significantly negatively correlated with the immune score and other immune cell subtypes in CC.

To clarify the potential mechanisms underlying the involvement of P4HA2 in the enhancement of immune cell infiltration, we calculated the correlations of P4HA2 with immune checkpoints (CD274, CTLA4, HAVCR2, LAG3, PDCD1, PDCD1LG2, SIGLEC15, and TIGIT) (Figure 6F). All of the eight immune checkpoints were significantly correlated with the P4HA2 expression in BRCA, COAD (colon adenocarcinoma), LGG, and PRAD. Furthermore, P4HA2 expression was positively correlated with immune checkpoints in the majority of cancers. Therefore, P4HA2 was not only correlated with the extent of immune infiltration, but potentially played a key role in regulating immune evasion.

### Correlation of P4HA2 Expression With Immune Infiltration

**FIGURE 7:**
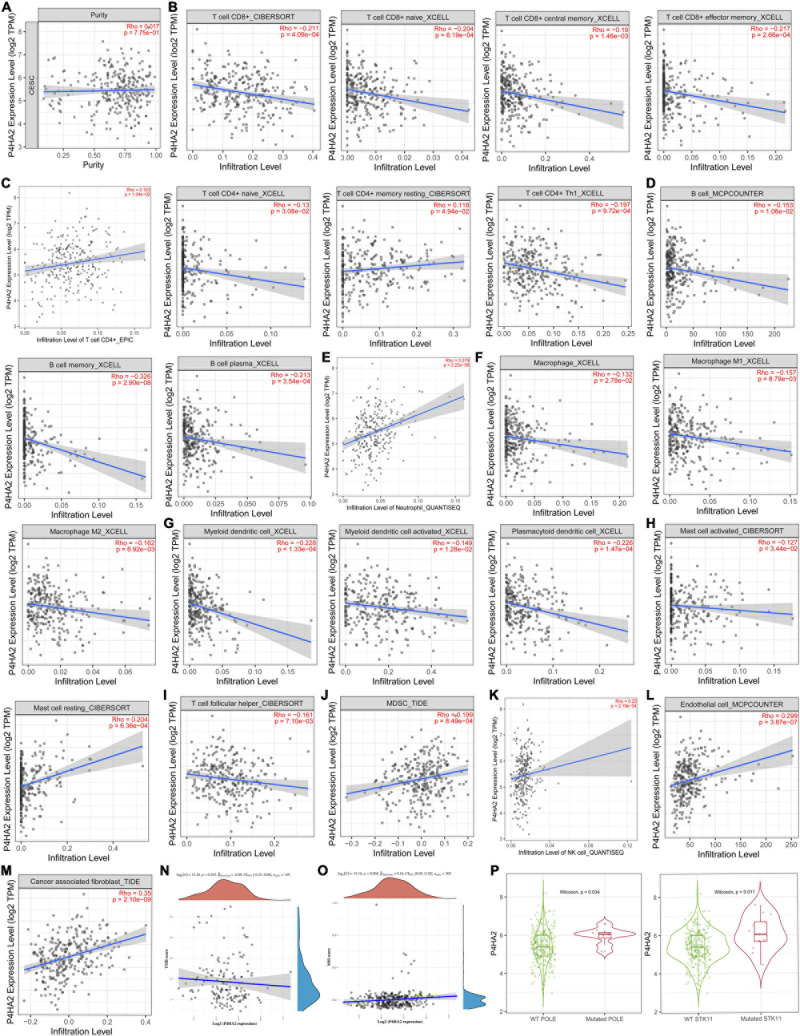
P4HA2 were noticeably correlated with immune infiltration. **(A–K)** The relationships between P4HA2 and immune cell infiltration levels calculated by TIMER database. **(L,M)** The relationships between endothelial cells, cancer associated fibroblast cells and P4HA2 expression. **(N,O)** The relationships between TMB, MSI, and P4HA2. **(P)** The differential expression between POLE/STK11 wild-type and POLE/STK11 mutation-type group for P4HA2.

To further explore the potential value of combined immunotherapy and targeting P4HA2 for the synergistic treatment of CC, we obtained the coefficient of P4HA2 and immune cells using TIMER database. No correlations was found between P4HA2 and tumor purity (Figure 7A). The results also revealed that P4HA2 was negatively correlated with CD8+T cells, naïve CD8+T cells, central memory CD8+T cells, effector memory CD8+T cells, naïve CD4+T cells, CD4+Th1 cells, B cells, memory B cells, macrophage (M0, M1, and M2), (activated) myeloid dendritic cells, plasmacytoid dendritic cells, activated mast cells, and follicular helper T cells (Figures 7B–D,F–I). On the contrary, P4HA2 expression was positively correlated with CD4+T cells, memory resting CD4+T cells, neutrophil cells, resting mast cells, MDSC cells, and NK cells (Figures 7C,E,H,J,K). Further information was available in the Supplementary Figures 2A–G. Moreover, P4HA2 positively correlated with endothelia cells (*r* = 0.299, *P* = 3.87e-07) and cancer associated fibroblast (CAF) cells (*r* = 0.35, *P* = 2.10e-09) (Figures 7L,M).

### P4HA2 Is Associated With the Microsatellite Instability and Immune Checkpoint

**FIGURE 8:**
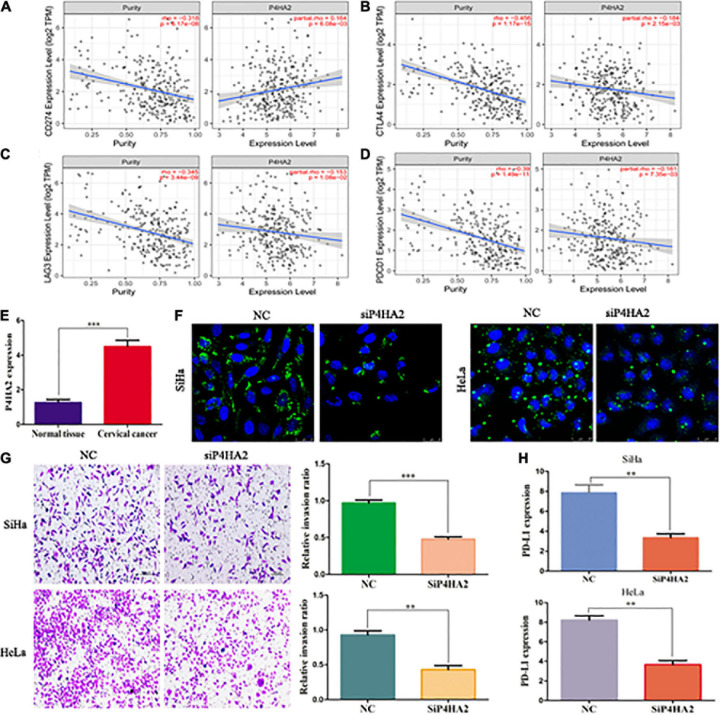
P4HA2 might promote CC metastasis through regulating the LDs accumulation and immune escape. **(A–D)** The relationships between P4HA2 and immune checkpoint genes. **(E)** The qRT-PCR results showed that P4HA2 was more highly expressed at the CC tissues than normal uterine cervical tissues. **(F)** LDs were detected by double staining with BODIPY 493/503 dye and Hoechst in the indicated cells. Scale bars, 25 um. **(G)** P4HA2 inhibited SiHa cells invasion by transwell assay. **(H)** The qRT-PCR analysis of PD-L1 expression in SiHa cells transfected with siP4HA2. **P < 0.01, ***P < 0.001.

Microsatellite instability (MSI), tumor mutational burden (TMB) and PDL-1 are the most important biomarkers for immune therapy in clinical practice (Mazloom et al., 2020). According to correlation analysis, P4HA2 was significantly positively correlated with MSI but not associated with TMB (Figures 7N,O). In addition, patients with POLE/STK11 mutations are more sensitive to immune checkpoint inhibitors (Skoulidis et al., 2018; Wang et al., 2019). P4HA2 was upregulated in POLE/STK11 mutation groups of CC patients (Figure 7P). Next, we analyzed the correlation between P4HA2 and immune checkpoint genes. P4HA2 was positively correlated with CD274 (PD-L1) and negatively correlated with CTLA4, LAG3, and PDCD1 (Figures 8A–D). However, P4HA2 was not significantly correlated with HAVCR2, PDCD1LG2, TIGIT, and SIGLEC15 (Supplementary Figure 2H).

### Knockdown of P4HA2 Might Inhibit Tumor Metastasis Through Regulating the Lipid Droplet Accumulation and Immune Escape

To further confirm the effects of P4HA2 in regulating immune microenvironment of CC, we firstly detected the expression levels of P4HA2 in CC tissues and normal tissues. Our data showed that P4HA2 was significantly higher in CC tissues compared with normal tissues (Figure 8E). Next, the transfection efficiencies of interference reagents for P4HA2 were confirmed by qRT-PCR (Supplementary Figure 2I). Knockdown of P4HA2 strongly decreased the number of LDs in CC cells, suggesting that P4HA2 modulated LD accumulation *in vitro* (Figure 8F). Transwell assays indicated that P4HA2 knockdown significantly inhibited the invasion ability of SiHa and HeLa cells (Figure 8G). Importantly, significant downregulation of PD-L1 was observed in CC cells after P4HA2 knockdown (Figure 8H). Thus, we reasoned that P4HA2 might inhibit CC metastasis through regulating tumor immune escape.

## Discussion

The appropriate strategy for treating CC depends on tumor stage at diagnosis, but the prognosis of patients with locally advanced or metastatic disease remains dismal (Pfaendler and Tewari, 2016). Immunotherapy as the new frontier of the anticancer treatment, is being an available option in treating this part of CC patients (Ventriglia et al., 2017). Here, for the first time, we identify the metabolic-related prognostic signature to provide an effective prognostic model of CC patients and potential predictive biomarkers of benefit from immunotherapy.

There is growing evidence that metabolism reprogramming has considered as a hallmark of cancer (Ward and Thompson, 2012). It is worth nothing that the Warburg effect may contribute to the early stages of tumor development. Instead, the lipid metabolism confers a selective advantage for tumor metastasis (Nath and Chan, 2016; Pascual et al., 2016). Our previous studies had been also confirmed that non-coding RNA mediated FA metabolism reprogramming could promote the process of lymph node metastasis (Shang et al., 2018). To establish an effective and simple scheme to explore the metabolic status and evaluate clinical outcomes of CC patients, we identify a prognostic MRGs model and patients with high risk scores exhibit a poor prognosis. Moreover, a nomograph composed of MRGs model and other clinical factors was constructed to better predict the OS. Our results illustrated that MRGs model could be applied as independent prognostic factors and indexes of immune infiltration status. Other study have found that immune score can also be used to evaluate the prognosis of CC patients (Chen et al., 2021). However, a larger sample size is needed to evaluate whether MRGs scores can substitute immune scores.

Oncogene-driven metabolism reprogramming can profoundly influence the TME to limit immune responses and put up more barriers to cancer therapy (Bader et al., 2020). And that’s why we focus on the potential of targeting metabolic pathways to improve the TME for favoring cancer immunotherapy. LDs as cytoplasmic lipid-rich organelles, have been verified to play an indispensable role in tumor pre-metastatic microenvironment (Shang et al., 2020). In our research, the MRGs were mainly enriched on the lipid metabolism pathway. Based on the MRGs model, the risk scores were inversely related to the immune score and TME scores. Meanwhile, memory B cells, follicular helper T cells, regulatory Tregs cells and resting mast cells were all negatively associated with the risk scores. Previous studies also showed that inhibition of glucose metabolism results in a drastic reduction of the frequency and number of follicular helper T cells (Choi et al., 2018). Thus, interventions targeting the metabolic pathways might reshape the immune state of the TME.

Collagen prolyl 4-hydroxylases (P4Hs) are located within the lumen of the endoplasmic reticulum and catalyzes post-translational formation of 4-hydroxyproline in -Xaa-Pro-Gly-sequences in collagens (Kivirikko and Pihlajaniemi, 1998). P4HA2 as one of the subtypes of collagen prolyl-4-hydroxylases α isoforms, was activated by HIF-1 to inducing extracellular matrix remodeling under hypoxic conditions and promoting cancer metastasis (Gilkes et al., 2013; Xiong et al., 2014). Our research indicated that P4HA2 was a MRG with independent prognostic value. Significant missense mutation in P4HA2 was observed, and different protein or mRNA expression levels were verified between the CC tissues and normal tissues. Consistent with the study of Cao et al. (2020), P4HA2 was markedly upregulated in CC tissues and functions as an oncogene in promoting cell metastasis by inducing epithelial-mesenchymal transition.

With the help of co-expression analysis, P4HA2 was predicted to be highly connected with multiple immune infiltration cells, including CD8+T cells, CD4+ T cells, B cells, macrophage cells (M0, M1, and M2), dendritic cells, mast cells, neutrophil cells, and NK cells. Moreover, CD8+T cells, memory activated CD4+T cells, resting NK cells and resting mast cells exhibited a significantly difference in evaluating lymph node metastasis. Our results were in accordance with previous research showing that infiltration with CD8+T cells was negatively associated with pelvic lymph node metastasis and predicted poor survival outcomes in CC patients (Ohno et al., 2020). CD8+T cells are preferred immune cells for targeting cancer. PD-1 and CTLA-4 can be targeted for relieving CD8+T cells exhaustion and thereby eliminating antigen-expressing cancer cells (Farhood et al., 2019). Approximately 96% patients with locally advanced CC expressed PD-L1 and tended to have a worse progression-free survival (Enwere et al., 2017). In this study, P4HA2 was negatively associated with CD8+T cells and positively correlated with CD274 (PD-L1) and CAF cells. As previous research illustrate, CAFs could specifically excluding CD8+ T cells from tumors to inhibit immune response and promoted immune evasion (Ford et al., 2020). Thus, P4HA2 might be a highly reliable predictive biomarkers to facilitate patient selection for immune checkpoint inhibitor-based therapies.

There were still several limitations to our research. First, the verification with another independent database was lacked. Second, although the LDs accumulation modulated by P4HA2 had been validate though BODIPY 493/503 staining, the further experimental exploration was still needed to confirm the potential mechanism and clinical utility. At the last, our work only reflect certain aspects of the immune infiltration from the perspective of metabolism reprogramming.

## Conclusion

In conclusion, we identified 5-MRGs model and a prognostic nomogram to predict survival of CC patients, which could reflect the immune infiltration status. Our nomogram including MRGs model may provide a reference tool for clinicians to guide follow-ups for CC patients. The hub gene-P4HA2 could modulate the LDs accumulation in CC cells and was closely associated with tumor infiltrating cells lymphocytes and immune checkpoint genes. This study provides a new and feasible method for evaluating the prognosis of CC and explores the potential value to navigate metabolic pathways to enhance anti-tumor immunity and immunotherapy.

## Data Availability Statement

The datasets presented in this study can be found in online repositories. The names of the repository/repositories and accession number(s) can be found below: GEO accession GSE173097, https://www.ncbi.nlm.nih.gov/geo/query/acc.cgi?acc=GSE173097.

## Ethics Statement

The studies involving human participants were reviewed and approved by the Ethical Committee of the Peking University Third Hospital approved this study. The patients/participants provided their written informed consent to participate in this study.

## Author Contributions

HG worked on design and conception of this study. JH processed the microarray data and revised the manuscript. CS collected the data, performed the data analysis, and drafted the manuscript. All authors read and approved the final manuscript.

## Conflict of Interest

The authors declare that the research was conducted in the absence of any commercial or financial relationships that could be construed as a potential conflict of interest.

## Footnotes

1 https://portal.gdc.cancer.gov/

2 https://bioinformatics.mdanderson.org/estimate/index.html

3 https://gdc.cancer.gov/about-data/publications/panimmune

4 https://cistrome.shinyapps.io/timer/

5 https://gtexportal.org/home/datasets

6 www.cbioportal.org

## Abbreviations

CC: cervical cancer
OS: overall survival
FA: fatty acid
TCGA: The Cancer Genome Atlas
MRG: metabolic-related gene
STR: short tandem repeat
qRT-PCR: quantitative real-time PCR
LASSO: least absolute shrinkage and selection operator
CI: confidence interval
TME: tumor microenvironment
TIMER: Tumor Immune Estimation Resource
HPA: Human Protein Atlas
KM: Kaplan–Meier analysis
GSEA: gene set enrichment analysis
DFS: disease-free survival
ROC: receiver operating characteristic
P4HA2: prolyl 4-hydroxylase subunit alpha 2
P4HA1: prolyl 4-hydroxylase subunit alpha 1
ABL2: ABL proto-oncogene 2
CYP4F12: cytochrome P450 family 4 subfamily F member 12
GLTP: glycolipid transfer protein
AUC: area under the curve
PTME: primary tumor microenvironment
IHC: immunohistochemistry
CAF: cancer associated fibroblast cells
MSI: microsatellite instability
TMB: tumor mutational burden
P4Has: collagen prolyl 4-hydroxylases
LDs: lipid droplets.
