# Supplementary material for: Identification of an Metabolic Related Risk Signature Predicts Prognosis in Cervical Cancer and Correlates With Immune Infiltration
Source: Front Cell Dev Biol. 2021 Jun 24;9:677831. doi: 10.3389/fcell.2021.677831 (PMC8264424; doi:10.3389/fcell.2021.677831)
Supplement: Supplementary file 1 [file Data_Sheet_1.pdf]

Supplementary Figure Legends

Supplementary Figure 1: The survival analysis for the 5-metabolic-related genes and the hierarchically-clustered heatmaps. (A) Kaplan–Meier (KM) curves for overall survival in patients of the TCGA database. Patients were divided into Low- and High-risk groups according to the median values. (B-C) The best cutoff value of MRGs score was identified to divide the CC patients. (D) ROC curve shows the sensitivity and specificity of MRGs model for predicting OS and DFS. (E-F) The best cutoff value of microenvironment score was identified to divide the CC patients. (G) Clustered heatmaps to explain the different expressions of 5-metabolic-related genes in various clinicopathological parameters.

Supplementary Figure 2: P4HA2 were noticeably correlated with immune cell infiltration. (A-G) The relationships between P4HA2 and the infiltration levels of immune cells calculated by the TIMER database. (H) The relationships between P4HA2 expression and immune checkpoint genes. (I) The efficiencies of P4HA2 knockdown.

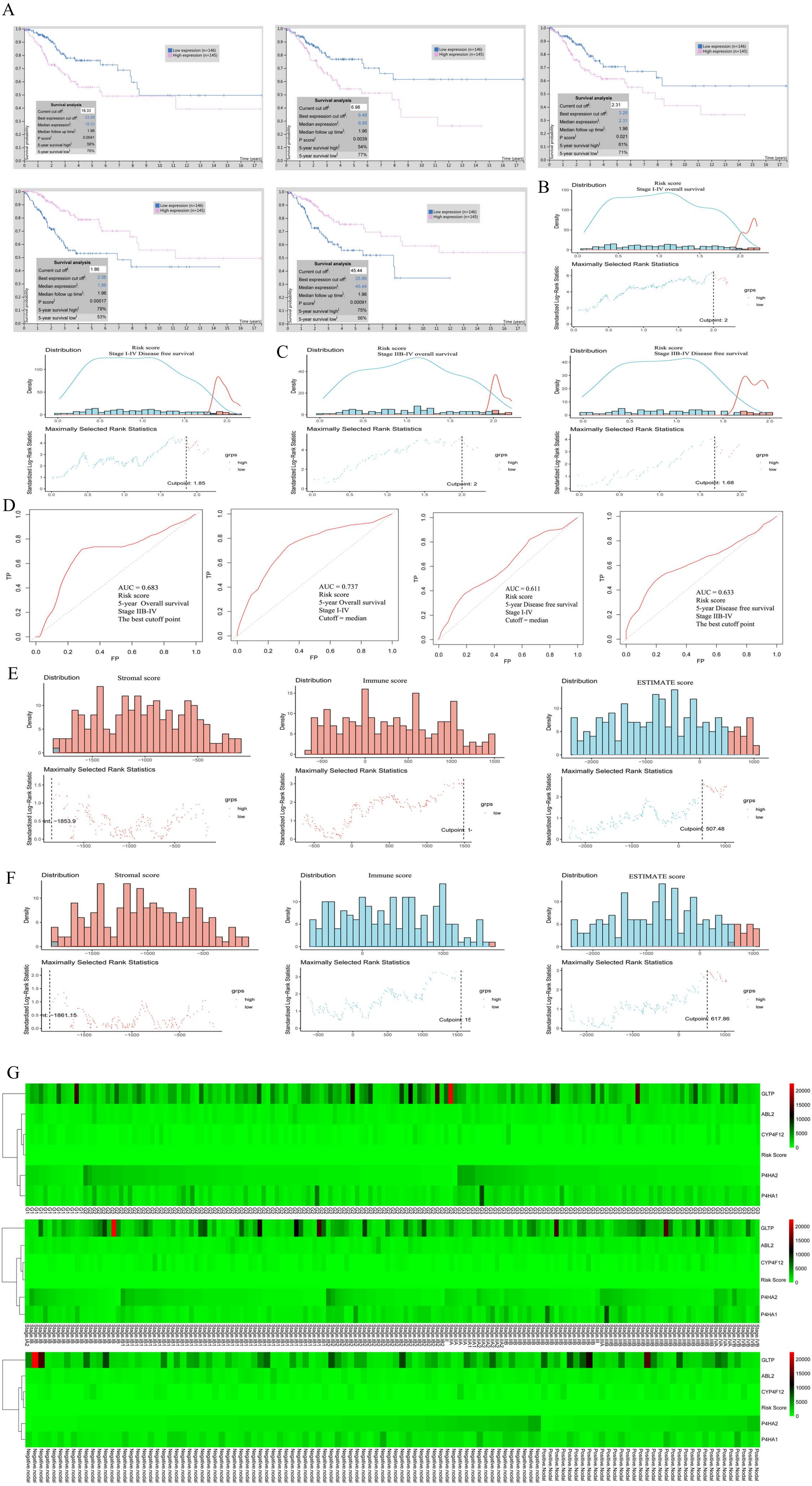

A
Survival analysis
Low expression (n=146)
High expression (n=145)
Survival probability
Time (years)
B
Distribution
Risk score
Stage I-IV overall survival
Maximally Selected Rank Statistics
Standardized Log-Rank Statistic
Cutpoint: 2
Stage I-IV Disease free survival
Cutpoint: 1.85
C
Stage IIB-IV overall survival
Stage IIB-IV Disease free survival
Cutpoint: 1.68
D
AUC = 0.683
Risk score
5-year Overall survival
Stage IIB-IV
The best cutoff point
AUC = 0.737
Risk score
5-year Overall survival
Stage I-IV
Cutoff = median
AUC = 0.611
Risk score
5-year Disease free survival
Stage I-IV
Cutoff = median
AUC = 0.633
Risk score
5-year Disease free survival
Stage IIB-IV
The best cutoff point
TP
FP
E
Stromal score
Immune score
ESTIMATE score
Cutpoint: 507.48
F
Cutpoint: 617.86
G
GLTP
ABL2
CYP4F12
Risk Score
P4HA2
P4HA1

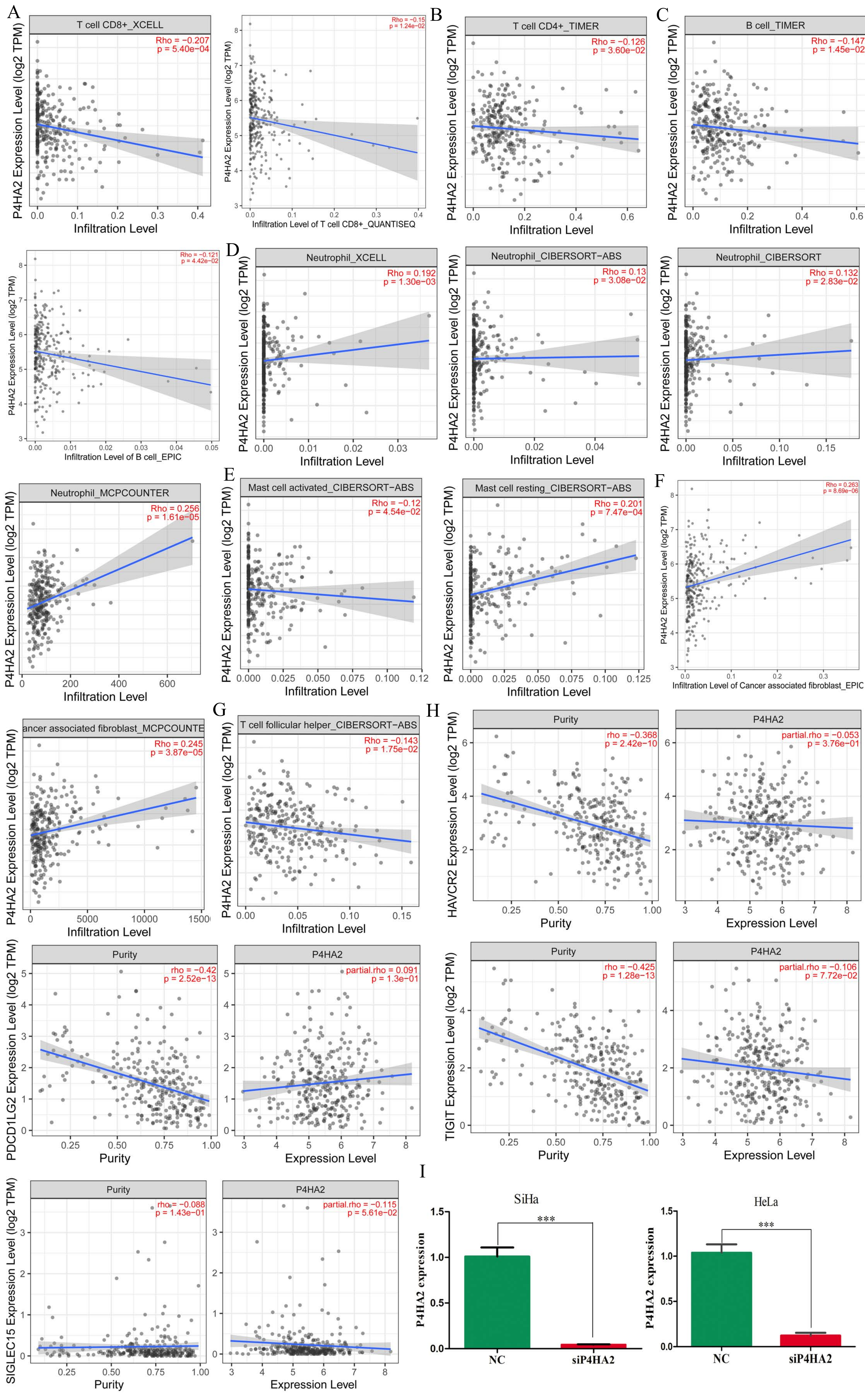
A
T cell CD8+_XCELL
Rho = −0.207
p = 5.40e-04
P4HA2 Expression Level (log2 TPM)
Infiltration Level
Rho = −0.15
p = 1.24e-02
Infiltration Level of T cell CD8+_QUANTISEQ
B
T cell CD4+_TIMER
Rho = −0.126
p = 3.60e-02
C
B cell_TIMER
Rho = −0.147
p = 1.45e-02
Rho = −0.121
p = 4.42e-02
Infiltration Level of B cell_EPIC
D
Neutrophil_XCELL
Rho = 0.192
p = 1.30e-03
Neutrophil_CIBERSORT−ABS
Rho = 0.13
p = 3.08e-02
Neutrophil_CIBERSORT
Rho = 0.132
p = 2.83e-02
Neutrophil_MCPCOUNTER
Rho = 0.256
p = 1.61e-05
E
Mast cell activated_CIBERSORT−ABS
Rho = −0.12
p = 4.54e-02
Mast cell resting_CIBERSORT−ABS
Rho = 0.201
p = 7.47e-04
F
Rho = 0.263
p = 8.69e-06
Infiltration Level of Cancer associated fibroblast_EPIC
ancer associated fibroblast_MCPCOUNTE
Rho = 0.245
p = 3.87e-05
G
T cell follicular helper_CIBERSORT−ABS
Rho = −0.143
p = 1.75e-02
H
Purity
rho = −0.368
p = 2.42e-10
HAVCR2 Expression Level (log2 TPM)
P4HA2
partial.rho = −0.053
p = 3.76e-01
Expression Level
Purity
rho = −0.42
p = 2.52e-13
PDCD1LG2 Expression Level (log2 TPM)
P4HA2
partial.rho = 0.091
p = 1.3e-01
Purity
rho = −0.425
p = 1.28e-13
TIGIT Expression Level (log2 TPM)
P4HA2
partial.rho = −0.106
p = 7.72e-02
Purity
rho = −0.088
p = 1.43e-01
SIGLEC15 Expression Level (log2 TPM)
P4HA2
partial.rho = −0.115
p = 5.61e-02
I
SiHa
HeLa
P4HA2 expression
***
NC
siP4HA2

Supplementary Table 1: The characterization of population samples from TCGA database

PATIENT_ID	AGE	HISTOLOGICAL DIAGNOSIS	KERATINIZATION SQUAMOUS CELL	GRADE	CLINICAL STAGE
TCGA-4J-AA1J	31	Cervical Squamous Cell	Non-keratinizing squamous cell	G3	Stage IB2
TCGA-BI-A0VR	53	Cervical Squamous Cell	Non-keratinizing squamous cell	G3	Stage IIIB
TCGA-BI-A0VS	48	Cervical Squamous Cell	Non-keratinizing squamous cell	G3	Stage IB
TCGA-BI-A20A	49	Cervical Squamous Cell	Non-keratinizing squamous cell	G3	Stage IB1
TCGA-C5-A0TN	21	Cervical Squamous Cell	Keratinizing squamous cell carcinoma	G3	Stage IB2
TCGA-C5-A1BE	64	Cervical Squamous Cell	Non-keratinizing squamous cell	G2	Stage IB2
TCGA-C5-A1BF	46	Cervical Squamous Cell	Keratinizing squamous cell carcinoma	G1	Stage IB
TCGA-C5-A1BI	31	Cervical Squamous Cell	[Not Available]	G2	Stage IIIB
TCGA-C5-A1BJ	34	Cervical Squamous Cell	[Not Available]	G2	Stage IIB
TCGA-C5-A1BK	36	Cervical Squamous Cell	Keratinizing squamous cell carcinoma	G2	Stage IB
TCGA-C5-A1BL	32	Cervical Squamous Cell	Non-keratinizing squamous cell	G2	Stage IB
TCGA-C5-A1BM	78	Cervical Squamous Cell	Non-keratinizing squamous cell	G2	Stage IB
TCGA-C5-A1BN	26	Cervical Squamous Cell	[Not Available]	G3	Stage IB2
TCGA-C5-A1BQ	65	Cervical Squamous Cell	Keratinizing squamous cell carcinoma	G2	Stage IIIB
TCGA-C5-A1M5	53	Cervical Squamous Cell	Keratinizing squamous cell carcinoma	G2	Stage IB
TCGA-C5-A1M6	55	Cervical Squamous Cell	[Not Available]	G3	Stage IIB
TCGA-C5-A1M7	37	Cervical Squamous Cell	Non-keratinizing squamous cell	G2	Stage IB
TCGA-C5-A1M8	43	Cervical Squamous Cell	[Not Available]	G2	Stage IB1
TCGA-C5-A1M9	46	Endocervical Type of	[Not Available]	G3	Stage IB1
TCGA-C5-A1ME	40	Endocervical Type of	[Not Available]	G1	Stage IB1
TCGA-C5-A1MF	49	Cervical Squamous Cell	Keratinizing squamous cell carcinoma	G2	Stage IB2
TCGA-C5-A1MH	71	Cervical Squamous Cell	Keratinizing squamous cell carcinoma	G3	Stage IIIB
TCGA-C5-A1MI	50	Cervical Squamous Cell	[Not Available]	G2	Stage IB1
TCGA-C5-A1MJ	61	Endocervical Type of	[Not Available]	G2	Stage IB1
TCGA-C5-A1MK	79	Cervical Squamous Cell	Keratinizing squamous cell carcinoma	G3	Stage IIIB
TCGA-C5-A1ML	49	Cervical Squamous Cell	[Not Available]	G3	Stage IB2
TCGA-C5-A1MN	42	Cervical Squamous Cell	Keratinizing squamous cell carcinoma	G2	Stage IIIB
TCGA-C5-A1MP	34	Cervical Squamous Cell	[Not Available]	G3	Stage IB2
TCGA-C5-A1MQ	35	Cervical Squamous Cell	[Not Available]	G2	Stage IIA
TCGA-C5-A2LS	37	Endocervical Adenocarcinoma of the Usual Type	[Not Available]	G1	Stage IB2
TCGA-C5-A2LT	38	Cervical Squamous Cell	Keratinizing squamous cell carcinoma	G3	Stage IB
TCGA-C5-A2LV	36	Cervical Squamous Cell	Non-keratinizing squamous cell	G3	Stage IB
TCGA-C5-A2LX	54	Cervical Squamous Cell	Keratinizing squamous cell carcinoma	G2	Stage IB1
TCGA-C5-A2LY	30	Cervical Squamous Cell	Non-keratinizing squamous cell	G2	Stage IB1
TCGA-C5-A2LZ	65	Cervical Squamous Cell	Keratinizing squamous cell carcinoma	G2	Stage IIIB
TCGA-C5-A2M1	37	Endocervical Type of	[Not Available]	G2	Stage IB1
TCGA-C5-A2M2	56	Endocervical Type of	[Not Available]	G2	Stage IB2
TCGA-C5-A3HD	51	Cervical Squamous Cell	[Not Available]	G2	Stage IIB
TCGA-C5-A3HE	44	Cervical Squamous Cell	Non-keratinizing squamous cell	G3	Stage IB2
TCGA-C5-A3HF	24	Mucinous Adenocarcinoma of Endocervical Type	[Not Available]	G2	Stage IB2
TCGA-C5-A3HL	76	Cervical Squamous Cell	Keratinizing squamous cell carcinoma	G2	Stage IB2
TCGA-C5-A7CG	55	Cervical Squamous Cell	Keratinizing squamous cell carcinoma	G2	Stage IB
TCGA-C5-A7CH	43	Cervical Squamous Cell	Keratinizing squamous cell carcinoma	G2	Stage IIB
TCGA-C5-A7CJ	42	Cervical Squamous Cell	Keratinizing squamous cell carcinoma	G2	Stage IIA
TCGA-C5-A7CK	58	Cervical Squamous Cell	Keratinizing squamous cell carcinoma	G2	Stage IVA
TCGA-C5-A7CL	48	Cervical Squamous Cell	Keratinizing squamous cell carcinoma	G2	Stage IIIB
TCGA-C5-A7CM	35	Endocervical Adenocarcinoma of the Usual Type	[Not Available]	G2	Stage IB2
TCGA-C5-A7CO	68	Cervical Squamous Cell	[Not Available]	G2	Stage IB2
TCGA-C5-A7UC	48	Cervical Squamous Cell	Non-keratinizing squamous cell	G3	Stage IB
TCGA-C5-A7UE	45	Cervical Squamous Cell	[Not Available]	G2	Stage IB1
TCGA-C5-A7UH	55	Cervical Squamous Cell	[Not Available]	G3	Stage IIIB
TCGA-C5-A7UI	42	Cervical Squamous Cell	Non-keratinizing squamous cell	G3	Stage IB1
TCGA-C5-A7X3	70	Cervical Squamous Cell	Non-keratinizing squamous cell	G2	Stage IIIB
TCGA-C5-A7X5	72	Cervical Squamous Cell	Keratinizing squamous cell carcinoma	G3	Stage IVB

TCGA-C5-A7X8	35	Mucinous Adenocarcinoma of Endocervical Type	[Not Available]	G2	Stage IB1
TCGA-C5-A7XC	26	Cervical Squamous Cell	[Not Available]	G2	Stage IB1
TCGA-C5-A8XH	39	Cervical Squamous Cell Carcinoma	Keratinizing squamous cell carcinoma	[Not Available	Stage IB1
TCGA-C5-A8XI	69	Cervical Squamous Cell	Non-keratinizing squamous cell	G3	Stage IB2
TCGA-C5-A8XJ	74	Cervical Squamous Cell Carcinoma	[Not Available]	[Not Available	Stage IB
TCGA-C5-A8XK	30	Cervical Squamous Cell Carcinoma	[Not Available]	G3	[Not Available]
TCGA-C5-A8YQ	79	Cervical Squamous Cell	[Not Available]	G2	Stage IB1
TCGA-C5-A8YR	56	Cervical Squamous Cell	Non-keratinizing squamous cell	G3	Stage IB
TCGA-C5-A8YT	36	Cervical Squamous Cell	[Not Available]	G3	Stage IB1
TCGA-C5-A8ZZ	41	Cervical Squamous Cell	Keratinizing squamous cell carcinoma	G2	Stage IIB
TCGA-C5-A901	44	Cervical Squamous Cell Carcinoma	Keratinizing squamous cell carcinoma	G2	[Not Available]
TCGA-C5-A902	35	Cervical Squamous Cell	[Not Available]	G3	Stage IB2
TCGA-C5-A905	37	Cervical Squamous Cell	Non-keratinizing squamous cell	G2	Stage IB
TCGA-C5-A907	47	Cervical Squamous Cell	[Not Available]	G2	Stage IB2
TCGA-DG-A2KH	25	Adenosquamous	[Not Available]	GX	Stage IB1
TCGA-DG-A2KJ	50	Cervical Squamous Cell	Non-keratinizing squamous cell	G3	Stage IIIB
TCGA-DG-A2KK	51	Cervical Squamous Cell	[Not Available]	G3	Stage IIIB
TCGA-DG-A2KL	53	Cervical Squamous Cell	Keratinizing squamous cell carcinoma	G1	Stage IIA
TCGA-DG-A2KM	46	Cervical Squamous Cell	[Not Available]	G2	Stage IB1
TCGA-DR-A0ZL	53	Cervical Squamous Cell	Non-keratinizing squamous cell	G3	Stage IB
TCGA-DR-A0ZM	61	Cervical Squamous Cell	Non-keratinizing squamous cell	G2	Stage IIIB
TCGA-DS-A0VK	45	Cervical Squamous Cell	Keratinizing squamous cell carcinoma	G3	Stage IB
TCGA-DS-A0VL	25	Cervical Squamous Cell	Non-keratinizing squamous cell	G2	Stage IB
TCGA-DS-A0VM	51	Cervical Squamous Cell	Non-keratinizing squamous cell	G3	Stage IB
TCGA-DS-A0VN	47	Cervical Squamous Cell	Keratinizing squamous cell carcinoma	G2	Stage IB
TCGA-DS-A1O9	75	Cervical Squamous Cell	Keratinizing squamous cell carcinoma	G3	Stage IVA
TCGA-DS-A1OA	77	Cervical Squamous Cell	Non-keratinizing squamous cell	G3	Stage IB
TCGA-DS-A1OB	45	Cervical Squamous Cell	Keratinizing squamous cell carcinoma	G2	Stage IB
TCGA-DS-A1OC	47	Cervical Squamous Cell	Keratinizing squamous cell carcinoma	G2	Stage IB
TCGA-DS-A1OD	49	Cervical Squamous Cell	Keratinizing squamous cell carcinoma	G3	Stage IB1
TCGA-DS-A3LQ	46	Cervical Squamous Cell	Non-keratinizing squamous cell	G3	Stage IIIB
TCGA-DS-A5RQ	80	Cervical Squamous Cell	Keratinizing squamous cell carcinoma	G2	Stage IB1
TCGA-DS-A7WF	41	Adenosquamous	Non-keratinizing squamous cell	G3	Stage IB2
TCGA-DS-A7WH	34	Mucinous Adenocarcinoma of Endocervical Type	Non-keratinizing squamous cell carcinoma	G2	Stage IB1
TCGA-DS-A7WI	43	Cervical Squamous Cell	Non-keratinizing squamous cell	G2	Stage IIA2
TCGA-EA-A1QS	46	Cervical Squamous Cell	Non-keratinizing squamous cell	G2	Stage IB1
TCGA-EA-A1QT	47	Cervical Squamous Cell	Non-keratinizing squamous cell	G2	Stage IB
TCGA-EA-A3HQ	60	Cervical Squamous Cell	Non-keratinizing squamous cell	G2	Stage II
TCGA-EA-A3HR	57	Cervical Squamous Cell	Non-keratinizing squamous cell	G2	Stage II
TCGA-EA-A3HS	35	Cervical Squamous Cell	Keratinizing squamous cell carcinoma	G1	Stage IB
TCGA-EA-A3HT	68	Cervical Squamous Cell	Keratinizing squamous cell carcinoma	G1	Stage IB
TCGA-EA-A3HU	43	Cervical Squamous Cell	Non-keratinizing squamous cell	G2	Stage II
TCGA-EA-A3QD	59	Cervical Squamous Cell	Non-keratinizing squamous cell	G3	Stage IIIB
TCGA-EA-A3QE	45	Cervical Squamous Cell	Non-keratinizing squamous cell	G2	Stage IB
TCGA-EA-A3Y4	40	Cervical Squamous Cell	Non-keratinizing squamous cell	G3	Stage IB
TCGA-EA-A410	51	Cervical Squamous Cell	Non-keratinizing squamous cell	G2	Stage IIA2
TCGA-EA-A411	50	Cervical Squamous Cell	Non-keratinizing squamous cell	G2	Stage IB1
TCGA-EA-A439	50	Cervical Squamous Cell	Non-keratinizing squamous cell	G3	Stage IIA1
TCGA-EA-A43B	43	Cervical Squamous Cell	Keratinizing squamous cell carcinoma	G2	Stage IB1
TCGA-EA-A44S	31	Cervical Squamous Cell	Non-keratinizing squamous cell	G2	Stage IIIB
TCGA-EA-A4BA	49	Endocervical Type of	[Not Available]	G2	Stage IB2
TCGA-EA-A50E	45	Cervical Squamous Cell	Non-keratinizing squamous cell	G2	Stage IVA
TCGA-EA-A556	38	Endometrioid Adenocarcinoma of Endocervix	[Not Available]	G3	Stage IB1

TCGA-EA-A5FO	59	Cervical Squamous Cell	Non-keratinizing squamous cell	G2	Stage IB1
TCGA-EA-A5O9	39	Cervical Squamous Cell	Non-keratinizing squamous cell	G2	Stage IB2
TCGA-EA-A5ZD	40	Cervical Squamous Cell	Non-keratinizing squamous cell	G2	Stage IB1
TCGA-EA-A5ZE	54	Cervical Squamous Cell	Non-keratinizing squamous cell	G3	Stage IB1
TCGA-EA-A5ZF	56	Cervical Squamous Cell	Non-keratinizing squamous cell	G2	Stage IB1
TCGA-EA-A6QX	49	Cervical Squamous Cell	Non-keratinizing squamous cell	G3	Stage IIIB
TCGA-EA-A78R	54	Cervical Squamous Cell	Non-keratinizing squamous cell	G2	Stage IB1
TCGA-EA-A97N	38	Cervical Squamous Cell	Non-keratinizing squamous cell	G2	Stage IB2
TCGA-EK-A2GZ	64	Cervical Squamous Cell	Non-keratinizing squamous cell	G2	Stage IIIB
TCGA-EK-A2H0	24	Cervical Squamous Cell	Non-keratinizing squamous cell	G3	Stage IIB
TCGA-EK-A2H1	20	Cervical Squamous Cell	Non-keratinizing squamous cell	G3	Stage IB2
TCGA-EK-A2IP	28	Cervical Squamous Cell	Non-keratinizing squamous cell	G3	Stage IB1
TCGA-EK-A2IR	48	Cervical Squamous Cell	Keratinizing squamous cell carcinoma	G3	Stage IB2
TCGA-EK-A2PG	88	Cervical Squamous Cell Carcinoma	[Not Available]	G3	[Not Available]
TCGA-EK-A2PI	44	Cervical Squamous Cell	[Not Available]	G2	Stage IIIB
TCGA-EK-A2PK	43	Cervical Squamous Cell	Non-keratinizing squamous cell	G3	Stage IB1
TCGA-EK-A2PL	36	Cervical Squamous Cell Carcinoma	Non-keratinizing squamous cell carcinoma	[Not Available	Stage IIIB
TCGA-EK-A2PM	81	Cervical Squamous Cell	Keratinizing squamous cell carcinoma	G3	Stage IIB
TCGA-EK-A2R7	45	Cervical Squamous Cell	[Not Available]	G3	Stage IB
TCGA-EK-A2R8	48	Cervical Squamous Cell	Keratinizing squamous cell carcinoma	G3	Stage IB2
TCGA-EK-A2R9	58	Cervical Squamous Cell	[Not Available]	G3	Stage IB1
TCGA-EK-A2RA	74	Cervical Squamous Cell	Non-keratinizing squamous cell	G3	Stage IIA2
TCGA-EK-A2RB	48	Cervical Squamous Cell	[Not Available]	G3	Stage IVB
TCGA-EK-A2RC	33	Cervical Squamous Cell	[Not Available]	G3	Stage IB1
TCGA-EK-A2RE	26	Cervical Squamous Cell	Keratinizing squamous cell carcinoma	G2	Stage IIA
TCGA-EK-A2RJ	51	Cervical Squamous Cell	Non-keratinizing squamous cell	G3	Stage IB2
TCGA-EK-A2RK	67	Cervical Squamous Cell	Non-keratinizing squamous cell	G3	Stage IA2
TCGA-EK-A2RL	32	Endocervical Type of	[Not Available]	G2	Stage IB
TCGA-EK-A2RM	40	Cervical Squamous Cell	[Not Available]	G3	Stage IB
TCGA-EK-A2RN	45	Cervical Squamous Cell	Keratinizing squamous cell carcinoma	G2	Stage IB1
TCGA-EK-A2RO	59	Cervical Squamous Cell	Keratinizing squamous cell carcinoma	G1	Stage IIB
TCGA-EK-A3GJ	51	Cervical Squamous Cell	Keratinizing squamous cell carcinoma	G3	Stage IB1
TCGA-EK-A3GK	33	Endocervical Type of	[Not Available]	G3	Stage IB1
TCGA-EK-A3GM	65	Endocervical Type of	[Not Available]	G2	Stage IIA
TCGA-EK-A3GN	47	Cervical Squamous Cell	[Not Available]	G3	Stage IIIB
TCGA-EX-A1H5	58	Cervical Squamous Cell	Non-keratinizing squamous cell	G3	Stage IIB
TCGA-EX-A1H6	38	Endocervical Type of	[Not Available]	G1	Stage IB1
TCGA-EX-A3L1	32	Cervical Squamous Cell	Non-keratinizing squamous cell	G3	Stage IIA1
TCGA-EX-A449	42	Endocervical Type of	[Not Available]	G1	Stage IVB
TCGA-EX-A69L	41	Cervical Squamous Cell	Non-keratinizing squamous cell	G3	Stage IB1
TCGA-EX-A69M	62	Cervical Squamous Cell	Non-keratinizing squamous cell	G3	Stage IB2
TCGA-EX-A8YF	44	Cervical Squamous Cell	Non-keratinizing squamous cell	G3	Stage IB1
TCGA-FU-A23K	28	Cervical Squamous Cell	Non-keratinizing squamous cell	G3	Stage IIIB
TCGA-FU-A23L	60	Cervical Squamous Cell	[Not Available]	G3	Stage IIA1
TCGA-FU-A2QG	29	Cervical Squamous Cell	Keratinizing squamous cell carcinoma	G2	Stage IB1
TCGA-FU-A3EO	55	Endocervical Type of	[Not Available]	G2	Stage IIB
TCGA-FU-A3HY	47	Cervical Squamous Cell	Non-keratinizing squamous cell	G2	Stage IB2
TCGA-FU-A3HZ	64	Cervical Squamous Cell	Non-keratinizing squamous cell	G3	Stage IIA2
TCGA-FU-A3NI	45	Cervical Squamous Cell	Keratinizing squamous cell carcinoma	G2	Stage IB1
TCGA-FU-A3TQ	55	Cervical Squamous Cell	Non-keratinizing squamous cell	G2	Stage IIIB
TCGA-FU-A3TX	78	Cervical Squamous Cell	Keratinizing squamous cell carcinoma	G3	Stage IB2
TCGA-FU-A3WB	43	Cervical Squamous Cell	Non-keratinizing squamous cell	G2	Stage IIA2
TCGA-FU-A3YQ	35	Cervical Squamous Cell	Non-keratinizing squamous cell	G1	Stage IB1
TCGA-FU-A40J	38	Endocervical Type of	[Not Available]	G3	Stage IIIB
TCGA-FU-A57G	49	Endocervical Type of	[Not Available]	G2	Stage IB2
TCGA-FU-A5XV	32	Cervical Squamous Cell	Non-keratinizing squamous cell	G3	Stage IIIB
TCGA-FU-A770	33	Endocervical Adenocarcinoma of the Usual Type	[Not Available]	G2	Stage IIIB

TCGA-GH-A9DA	27	Cervical Squamous Cell	Non-keratinizing squamous cell	G3	Stage IB1
TCGA-HG-A2PA	38	Cervical Squamous Cell	Non-keratinizing squamous cell	G2	Stage IB2
TCGA-HM-A3JJ	40	Cervical Squamous Cell	Non-keratinizing squamous cell	G3	Stage IB1
TCGA-HM-A3JK	64	Cervical Squamous Cell	[Not Available]	G3	Stage IIA2
TCGA-HM-A4S6	51	Cervical Squamous Cell	[Not Available]	G3	Stage IIIB
TCGA-HM-A6W2	34	Adenosquamous	[Not Available]	G3	Stage IVB
TCGA-IR-A3L7	37	Cervical Squamous Cell	[Not Available]	G3	Stage IB1
TCGA-IR-A3LA	60	Endometrioid Adenocarcinoma of Endocervix	[Not Available]	G3	Stage IB1
TCGA-IR-A3LB	53	Endocervical Type of	[Not Available]	G3	Stage IB1
TCGA-IR-A3LC	40	Cervical Squamous Cell	[Not Available]	G3	Stage IB1
TCGA-IR-A3LF	64	Endocervical Adenocarcinoma of the Usual Type	[Not Available]	G2	Stage IB1
TCGA-IR-A3LH	49	Cervical Squamous Cell	[Not Available]	G4	Stage IIA1
TCGA-IR-A3LI	48	Endocervical Type of	[Not Available]	G2	Stage IVB
TCGA-IR-A3LK	69	Cervical Squamous Cell	[Not Available]	G3	Stage IB2
TCGA-IR-A3LL	60	Cervical Squamous Cell	[Not Available]	G2	Stage IB1
TCGA-JW-A5VG	35	Cervical Squamous Cell	Non-keratinizing squamous cell	G3	Stage IIA
TCGA-JW-A5VH	53	Cervical Squamous Cell	Non-keratinizing squamous cell	G2	Stage IVB
TCGA-JW-A5VI	45	Cervical Squamous Cell	Non-keratinizing squamous cell	G3	Stage IIB
TCGA-JW-A5VJ	56	Cervical Squamous Cell	Non-keratinizing squamous cell	G3	Stage IIB
TCGA-JW-A5VK	43	Cervical Squamous Cell	Non-keratinizing squamous cell	G3	Stage IB2
TCGA-JW-A5VL	37	Cervical Squamous Cell	Non-keratinizing squamous cell	G1	Stage IB2
TCGA-JW-A69B	44	Endocervical Type of	[Not Available]	GX	Stage IB2
TCGA-JW-A852	42	Cervical Squamous Cell	Non-keratinizing squamous cell	G2	Stage IIB
TCGA-JW-AAVH	46	Cervical Squamous Cell	Non-keratinizing squamous cell	G2	Stage IB1
TCGA-JX-A3PZ	25	Cervical Squamous Cell	Keratinizing squamous cell carcinoma	G2	Stage IB
TCGA-JX-A3Q0	63	Cervical Squamous Cell	Keratinizing squamous cell carcinoma	G2	Stage III
TCGA-JX-A3Q8	40	Endocervical Type of	[Not Available]	G3	Stage IB1
TCGA-JX-A5QV	37	Cervical Squamous Cell	Keratinizing squamous cell carcinoma	G3	Stage IB1
TCGA-LP-A4AU	35	Cervical Squamous Cell	[Not Available]	G3	Stage IIIB
TCGA-LP-A4AV	63	Cervical Squamous Cell	[Not Available]	G2	Stage IB
TCGA-LP-A4AW	52	Cervical Squamous Cell	Keratinizing squamous cell carcinoma	G1	Stage IA
TCGA-LP-A4AX	45	Cervical Squamous Cell	[Not Available]	G3	Stage IB1
TCGA-LP-A5U2	30	Endocervical Adenocarcinoma of the Usual Type	[Not Available]	G3	Stage IIIB
TCGA-LP-A5U3	40	Cervical Squamous Cell	Non-keratinizing squamous cell	G3	Stage IB1
TCGA-LP-A7HU	53	Endocervical Type of	[Not Available]	G3	Stage II
TCGA-MA-AA3W	54	Cervical Squamous Cell	Non-keratinizing squamous cell	G3	Stage IB1
TCGA-MA-AA3X	50	Cervical Squamous Cell	Non-keratinizing squamous cell	G2	Stage IIIB
TCGA-MA-AA3Y	48	Cervical Squamous Cell	Keratinizing squamous cell carcinoma	G3	Stage IB1
TCGA-MA-AA3Z	43	Cervical Squamous Cell	Non-keratinizing squamous cell	GX	Stage IB2
TCGA-MA-AA41	33	Cervical Squamous Cell	Non-keratinizing squamous cell	G2	Stage IIB
TCGA-MA-AA42	75	Cervical Squamous Cell	Non-keratinizing squamous cell	G3	Stage IIB
TCGA-MA-AA43	48	Cervical Squamous Cell	Non-keratinizing squamous cell	G3	Stage IIIB
TCGA-MU-A51Y	27	Cervical Squamous Cell	Non-keratinizing squamous cell	G2	Stage IIA1
TCGA-MU-A5YI	60	Cervical Squamous Cell	Non-keratinizing squamous cell	G2	Stage IA1
TCGA-MU-A8JM	46	Cervical Squamous Cell	Non-keratinizing squamous cell	G2	Stage IB1
TCGA-MY-A5BD	62	Cervical Squamous Cell	[Not Available]	G1	Stage IIB
TCGA-MY-A5BE	42	Cervical Squamous Cell	[Not Available]	G3	Stage IB1
TCGA-MY-A5BF	68	Cervical Squamous Cell	[Not Available]	G1	Stage IIA2
TCGA-MY-A913	28	Cervical Squamous Cell	[Not Available]	G3	Stage IIA
TCGA-PN-A8MA	43	Cervical Squamous Cell	Non-keratinizing squamous cell	G3	Stage IIB
TCGA-Q1-A5R1	32	Mucinous Adenocarcinoma of Endocervical Type	Non-keratinizing squamous cell carcinoma	G2	Stage IB1
TCGA-Q1-A5R2	64	Cervical Squamous Cell Carcinoma	Non-keratinizing squamous cell carcinoma	G3	[Not Available]
TCGA-Q1-A5R3	56	Cervical Squamous Cell Carcinoma	Non-keratinizing squamous cell carcinoma	G2	[Not Available]
TCGA-Q1-A6DT	55	Cervical Squamous Cell	Non-keratinizing squamous cell	GX	Stage I

TCGA-Q1-A6DV	36	Mucinous Adenocarcinoma of Endocervical Type	Non-keratinizing squamous cell carcinoma	G2	Stage IB1
TCGA-Q1-A6DW	44	Cervical Squamous Cell	Non-keratinizing squamous cell	GX	Stage I
TCGA-Q1-A73O	37	Cervical Squamous Cell	Non-keratinizing squamous cell	GX	Stage IB2
TCGA-Q1-A73P	45	Endocervical Type of	[Not Available]	G1	Stage IB1
TCGA-Q1-A73Q	46	Cervical Squamous Cell	Keratinizing squamous cell carcinoma	GX	Stage I
TCGA-Q1-A73R	45	Endocervical Type of	[Not Available]	GX	Stage I
TCGA-Q1-A73S	33	Adenosquamous	Non-keratinizing squamous cell	G2	Stage IB1
TCGA-R2-A69V	42	Cervical Squamous Cell	Non-keratinizing squamous cell	G3	Stage IB
TCGA-RA-A741	34	Cervical Squamous Cell	Non-keratinizing squamous cell	GX	Stage IIB
TCGA-UC-A7PD	21	Cervical Squamous Cell	Keratinizing squamous cell carcinoma	G2	Stage IB
TCGA-UC-A7PF	44	Cervical Squamous Cell	[Not Available]	G2	Stage IB1
TCGA-UC-A7PG	44	Cervical Squamous Cell	Non-keratinizing squamous cell	G1	Stage IIIB
TCGA-UC-A7PI	44	Endometrioid Adenocarcinoma of Endocervix	[Not Available]	G1	Stage IB1
TCGA-VS-A8EB	41	Cervical Squamous Cell	[Not Available]	GX	Stage IIIB
TCGA-VS-A8EC	55	Cervical Squamous Cell	[Not Available]	G2	Stage IIIB
TCGA-VS-A8EG	36	Cervical Squamous Cell	[Not Available]	GX	Stage IB1
TCGA-VS-A8EH	56	Cervical Squamous Cell	[Not Available]	G2	Stage IIIB
TCGA-VS-A8EI	38	Cervical Squamous Cell	[Not Available]	GX	Stage IIB
TCGA-VS-A8EJ	60	Cervical Squamous Cell	[Not Available]	G3	Stage IIB
TCGA-VS-A8EK	65	Cervical Squamous Cell	[Not Available]	G2	Stage IVA
TCGA-VS-A8EL	38	Cervical Squamous Cell	[Not Available]	G3	Stage IIB
TCGA-VS-A8Q8	26	Cervical Squamous Cell	[Not Available]	G2	Stage IB
TCGA-VS-A8Q9	79	Cervical Squamous Cell	[Not Available]	G2	Stage IB1
TCGA-VS-A8QA	44	Cervical Squamous Cell	[Not Available]	GX	Stage IB1
TCGA-VS-A8QC	51	Cervical Squamous Cell	[Not Available]	G2	Stage IVA
TCGA-VS-A8QF	42	Cervical Squamous Cell	[Not Available]	G2	Stage IIB
TCGA-VS-A8QH	76	Mucinous Adenocarcinoma of Endocervical Type	[Not Available]	G2	Stage IB1
TCGA-VS-A8QM	47	Cervical Squamous Cell	[Not Available]	GX	Stage IVB
TCGA-VS-A94W	39	Cervical Squamous Cell	[Not Available]	G2	Stage IIB
TCGA-VS-A94X	40	Cervical Squamous Cell	[Not Available]	G2	Stage IIB
TCGA-VS-A94Y	47	Cervical Squamous Cell	[Not Available]	GX	Stage IIB
TCGA-VS-A94Z	38	Cervical Squamous Cell	[Not Available]	G2	Stage IIB
TCGA-VS-A950	42	Cervical Squamous Cell	[Not Available]	G3	Stage IIIA
TCGA-VS-A952	66	Mucinous Adenocarcinoma of Endocervical Type	[Not Available]	G2	Stage IB1
TCGA-VS-A953	63	Cervical Squamous Cell	[Not Available]	GX	Stage IVA
TCGA-VS-A954	67	Cervical Squamous Cell	[Not Available]	G2	Stage IIIB
TCGA-VS-A957	64	Cervical Squamous Cell	[Not Available]	G3	Stage IB1
TCGA-VS-A958	46	Cervical Squamous Cell	[Not Available]	G2	Stage IIB
TCGA-VS-A959	76	Cervical Squamous Cell	[Not Available]	G3	Stage IIB
TCGA-VS-A9U5	57	Cervical Squamous Cell	[Not Available]	G3	Stage IIB
TCGA-VS-A9U6	52	Cervical Squamous Cell	Non-keratinizing squamous cell	GX	Stage IVB
TCGA-VS-A9U7	30	Cervical Squamous Cell	[Not Available]	G3	Stage IB1
TCGA-VS-A9UB	54	Cervical Squamous Cell	[Not Available]	G3	Stage IIB
TCGA-VS-A9UC	32	Cervical Squamous Cell	[Not Available]	G2	Stage IIB
TCGA-VS-A9UD	73	Cervical Squamous Cell	Keratinizing squamous cell carcinoma	G2	Stage IIIA
TCGA-VS-A9UH	53	Cervical Squamous Cell	[Not Available]	GX	Stage IVA
TCGA-VS-A9UI	76	Cervical Squamous Cell	[Not Available]	GX	Stage IIB
TCGA-VS-A9UJ	55	Cervical Squamous Cell	[Not Available]	GX	Stage IIB
TCGA-VS-A9UL	79	Cervical Squamous Cell	[Not Available]	G3	Stage IIIB
TCGA-VS-A9UM	39	Cervical Squamous Cell	[Not Available]	G2	Stage IVB
TCGA-VS-A9UO	43	Mucinous Adenocarcinoma of Endocervical Type	[Not Available]	G2	Stage IIB
TCGA-VS-A9UP	43	Mucinous Adenocarcinoma of Endocervical Type	[Not Available]	G3	Stage IIA
TCGA-VS-A9UQ	32	Mucinous Adenocarcinoma of Endocervical Type	[Not Available]	G2	Stage IB

TCGA-VS-A9UR	53	Mucinous Adenocarcinoma of Endocervical Type	[Not Available]	GX	Stage IIA
TCGA-VS-A9UT	72	Mucinous Adenocarcinoma of Endocervical Type	[Not Available]	G3	Stage IB
TCGA-VS-A9UU	42	Cervical Squamous Cell	[Not Available]	G1	Stage IIB
TCGA-VS-A9UV	74	Cervical Squamous Cell	[Not Available]	GX	Stage IVA
TCGA-VS-A9UY	29	Cervical Squamous Cell	Non-keratinizing squamous cell	G2	Stage IVB
TCGA-VS-A9UZ	61	Mucinous Adenocarcinoma of Endocervical Type	[Not Available]	G2	Stage IB1
TCGA-VS-A9V0	58	Mucinous Adenocarcinoma of Endocervical Type	[Not Available]	G3	Stage IB
TCGA-VS-A9V1	46	Mucinous Adenocarcinoma of Endocervical Type	[Not Available]	G2	Stage IVB
TCGA-VS-A9V2	29	Cervical Squamous Cell	[Not Available]	G2	Stage IB1
TCGA-VS-A9V3	62	Cervical Squamous Cell	[Not Available]	G3	Stage IVB
TCGA-VS-A9V4	63	Mucinous Adenocarcinoma of Endocervical Type	[Not Available]	G2	Stage IVA
TCGA-VS-A9V5	50	Mucinous Adenocarcinoma of Endocervical Type	[Not Available]	G2	Stage IIB
TCGA-VS-AA62	51	Cervical Squamous Cell	[Not Available]	G2	Stage IIB
TCGA-WL-A834	57	Cervical Squamous Cell Carcinoma	Non-keratinizing squamous cell carcinoma	G3	[Not Available]
TCGA-XS-A8TJ	41	Cervical Squamous Cell	Non-keratinizing squamous cell	G2	Stage IB1
TCGA-ZJ-A8QO	73	Cervical Squamous Cell Carcinoma	Non-keratinizing squamous cell carcinoma	[Not Available	[Not Available]
TCGA-ZJ-A8QQ	24	Cervical Squamous Cell	Non-keratinizing squamous cell	GX	Stage IIB
TCGA-ZJ-A8QR	38	Cervical Squamous Cell	Keratinizing squamous cell carcinoma	GX	Stage I
TCGA-ZJ-AAX4	85	Cervical Squamous Cell	Non-keratinizing squamous cell	G3	Stage II
TCGA-ZJ-AAX8	58	Cervical Squamous Cell	Non-keratinizing squamous cell	G2	Stage IIIB
TCGA-ZJ-AAXA	64	Cervical Squamous Cell	Non-keratinizing squamous cell	G2	Stage IB1
TCGA-ZJ-AAXB	42	Endocervical Adenocarcinoma of the Usual Type	[Not Available]	G3	Stage IB2
TCGA-ZJ-AAXD	35	Cervical Squamous Cell	Non-keratinizing squamous cell	G2	Stage IIIB
TCGA-ZJ-AAXF	62	Cervical Squamous Cell	Keratinizing squamous cell carcinoma	G3	Stage IIB
TCGA-ZJ-AAXI	67	Cervical Squamous Cell	Non-keratinizing squamous cell	G2	Stage IIB
TCGA-ZJ-AAXJ	43	Cervical Squamous Cell Carcinoma	Keratinizing squamous cell carcinoma	[Not Available	Stage IIB
TCGA-ZJ-AAXN	34	Cervical Squamous Cell Carcinoma	Non-keratinizing squamous cell carcinoma	[Not Available	Stage IB2
TCGA-ZJ-AAXT	54	Cervical Squamous Cell	Non-keratinizing squamous cell	G2	Stage IIIB
TCGA-ZJ-AAXU	51	Cervical Squamous Cell	Non-keratinizing squamous cell	G2	Stage IIB
TCGA-ZJ-AB0H	48	Cervical Squamous Cell Carcinoma	Non-keratinizing squamous cell carcinoma	[Not Available	Stage IIIB
TCGA-ZJ-AB0I	25	Cervical Squamous Cell Carcinoma	Keratinizing squamous cell carcinoma	[Not Available	Stage IIB
TCGA-ZX-AA5X	64	Cervical Squamous Cell	Non-keratinizing squamous cell	G2	Stage IIIB
